# A Longitudinal Study of Child Maltreatment and Mental Health Predictors of Admission to Psychiatric Residential Treatment Facilities

**DOI:** 10.3390/ijerph14101141

**Published:** 2017-09-28

**Authors:** Roderick A. Rose, Paul Lanier

**Affiliations:** School of Social Work, University of North Carolina at Chapel Hill, Chapel Hill, NC 27599, USA; planier@email.unc.edu

**Keywords:** child abuse, mental health services, child welfare

## Abstract

The child welfare system is an access point for children’s mental health services. Psychiatric residential treatment facilities (PRTFs) are the most restrictive, and most expensive setting for children to receive long-term care. Given the high rates of behavioral health concerns among maltreated children in out-of-home care, research is needed to examine the factors that predict entry in PRTFs among children investigated for maltreatment. This exploratory study used cross-sector administrative records linked across multiple systems, including child welfare records and Medicaid claims, from a single state over a five-year period (*n* = 105,982). Cox proportional hazards modeling was used to predict entry into a PRTF. After controlling for many factors, PRTF entry was predicted by diagnosis code indicating a trauma-related condition, antipsychotic medication prescriptions, and entry into lower levels of out-of-home care, supporting the view that youth are admitted to PRTFs largely due to clinical need. However, PRTF admission is also associated with characteristics of their experiences with the social service system, primarily foster care placement stability and permanency. Implications for practice and research are discussed.

## 1. Introduction

Children who experience abuse and neglect (maltreatment) are at greater risk for behavioral health concerns compared to children who do not experience this adverse childhood experience [1,2,3,4,5,6,7]. Also, children with behavioral health disorders are at greater risk for experiencing child maltreatment [8]. Therefore, integration of the child welfare and behavioral health care service systems is necessary to deliver interventions to children who have experienced trauma from maltreatment, but also to prevent future maltreatment by helping families appropriately manage and support children with challenging behavioral health conditions.

Maltreated children who receive services through the child welfare system may be placed in an out-of-home foster care setting. Children who enter foster care are a relatively small proportion of all children in families investigated for maltreatment. In 2014, there were 702,000 officially substantiated victims of child maltreatment and about one in five (23%) received foster care services [9]. At the end of fiscal year 2015, there were 427,910 children in foster care in the United States [10]. Identifying and maintaining a safe, permanent home is the goal of foster care services, typically through reunification or adoption. However, many children stay in foster care for long periods of time (median *=* 12.6 months) and move through multiple placement types and settings [10]. Out-of-home placement settings exist across a continuum, with federal law requiring that a child be placed in the “least restrictive (most family like)” setting (42 U.S. Code 675(5)). Further, the Individuals with Disabilities Education Act states that children with disabilities (which includes serious emotional disturbance), must receive special education services in the “least restrictive environment” to the maximum extent possible (20 U.S. Code 1412(5)).

Once removed from the home, the most restrictive child welfare setting for a child to receive care is in residential or congregate care. Most children are placed in foster family homes with a relative (30%) or non-relative (45%), but many children are placed in congregate care settings including group homes (6%) and institutions (8%) [10]. Although placement in congregate care has decreased overall for children in foster care, children with behavioral health concerns are more likely to enter congregate care settings [11]. Arguably the most restrictive institutional out-of-home care setting, psychiatric residential treatment facilities (PRTFs) are a necessary component of the care continuum, particularly following crisis stabilization for acute behavioral health concerns. However, as long-term care, not only are PRTFs restrictive, they are a costly care setting and have limited evidence of benefits beyond what could be offered in home or community-based care [12]. Given these concerns, it is critical to identify what factors are associated with PRTF entry to determine whether placement is driven by mainly clinical need and the extent to which other factors play a role.

The purpose of this study was to examine predictors of PRTF entry among children investigated for child maltreatment. This study was motivated by concerns in a state where PRTF expenditures had almost doubled in two years ($50 million in 2009 to $97 million in 2011) as the number of youth in PRTFs also doubled [13]. This increased use of PRTFs and associated Medicaid expenditures has been a concern at the federal level as well [12]. However, few studies have examined predictors of PRTF entry, particularly among maltreated children receiving child welfare services. Consistent with the behavioral model of healthcare utilization, we conceptualize use of PRTFs, like use of other healthcare services, as composed of three determinants: predisposing factors, enabling factors, and need [14]. Using linked, longitudinal administrative records, the current study sought to examine the predictors of PRTF entry among a statewide sample of children in families investigated for maltreatment. This knowledge will provide additional evidence to ultimately inform policy discussions regarding the best strategies to ensure children receive care in the most appropriate settings.

### 1.1. Background

As defined by Centers for Medicare and Medicaid Services (CMS), a psychiatric residential treatment facility (PRTF) is a non-hospital facility that provides inpatient services to Medicaid-eligible individuals under the age of 21 years. In 2015, there were 384 PRTFs in the United States, as defined by CMS [15]. PRTFs must be accredited and are bound by a variety of state and federal regulations (e.g., service provision under the direction of a physician and an individual plan of care developed by an interdisciplinary treatment team). PRTFs are highly-restrictive institutions that provide a setting for mental health service delivery intended for children suffering from serious emotional disturbances and behavioral disorders that cannot be managed with home- or community-based services. For an individual to receive PRTF services, a treatment team must certify that “ambulatory care resources available in the community do not meet treatment needs” (42 C.F.R. §441.152). PRTFs are intended to be a resource available to children who need intensive inpatient medical supervision in a highly-controlled environment. These treatment settings provide specialized interventions, room and board, and 24 h supervision, representing an often necessary but extremely costly method of delivering mental health care [16]. The added cost and restrictions may not, on average, benefit children who are admitted relative to their peers treated in less-restrictive and less-costly family and community settings [17].

### 1.2. Mental Health Service Need and Use among Maltreated Children

Several studies have explored the experience of children who overlap in the child welfare and mental health service systems. The National Survey of Child and Adolescent Well-Being (NSCAW) was the first nationally representative sample of children who were subject to reports of maltreatment investigated by child welfare agencies. In addition to information relating to the experience of child maltreatment, the need for mental health services and mental health service use was also assessed [5]. Study estimates suggest that among the 1.7 million children were investigated as possible victims of maltreatment during the time of NSCAW, 814,300 had a substantial need for mental health services but only 192,175 received services. NSCAW found high overall need and use of mental health services among maltreated children. However, when this need for services is unmet in community-based care, children remain placed in restrictive placements settings.

### 1.3. Prior Studies Examining Predictors of PRTF Entry

PRTFs are frequently grouped with less restrictive care settings such as congregate or residential treatment care including substance abuse treatment centers [18], but recent research using Medicaid reimbursement data shows that youth with clinical needs such as major depression, affective psychoses, and conduct disorders were commonly treated in PRTFs [16]. These studies, although demonstrating a link between behavioral health needs and PRTF admission in the population served by Child Protective Services (CPS) do not rule out that some of these needs emerge from the difficulties these youth encounter once they are being served by this system. Statistically, this means that after accounting for clinical need, there may yet be independent associations between PRTF admission and other predisposing and enabling characteristics such as experiences related to child maltreatment, foster care, and other services (i.e., Temporary Assistance for Needy Families (TANF)). For example, a recent study using a sample consisting largely of youth with foster care histories (96% had a prior out-of-home placement), found four latent classes of youth, all of whom had severe behavioral and emotional disturbances and histories of maltreatment, but who were distinguished largely by family strengths [19].

For youth in foster care, behavioral health care may both lead to and derive from placement instability [20]. Alternatively, youth who are placed with relatives, and particularly those placed more quickly in kinship settings, tend to have less intensive behavioral health needs [21]. It is not clear the role that economic well-being plays in PRTF admission. Recent research using survey data from Illinois suggests an association between material hardship and child maltreatment [22], suggesting a potentially greater role for government income maintenance services, such as TANF, among families investigated by CPS. Involvement with TANF has been shown to be associated with PRTF admission, after controlling for involvement in a CPS investigation or foster care [23]. However, it is not known if receipt of TANF, as an indicator of economic well-being, is independently associated with PRTF admission after controlling for youths’ behavioral health histories. Receipt of TANF can also be a proxy indicator of low-income status, given that program eligibility is means-tested.

The current study builds on a prior analysis of PRTF entry in one state in several important ways [23]. First, the prior study examined system contact broadly; the current analysis includes additional data that allows for a more detailed examination of children’s experiences within these systems. Second, the current study includes specific information regarding behavioral health care service utilization, mental health diagnosis, and prescription drug records allowing greater examination of the use of less restrictive services prior to PRTF entry and indicators of service need. Our objective is to help to uncover the extent to which PRTF admission is a response to clinical need versus other potentially correlated factors typical of youth served by CPS. This study brings together and links data at the individual child level using several sources of administrative data that document provision of these public services; these are the “big data” of social services.

### 1.4. Research Questions

Based on the limited prior research examining PRTF admission among children from families investigated for maltreatment, we conducted an exploratory study that sought to identify independent associations between child maltreatment, foster care, TANF receipt, and PRTF admission, after accounting for clinical need as demonstrated by behavioral and mental health characteristics. We specified three research questions pertaining to these children:

(1) What behavioral health characteristics, including diagnosis associated with trauma, behavioral health services utilization (family, group, and individual therapy; care in residential service settings; psychiatric services; and drug/alcohol services), and psychotropic and antipsychotic drug prescriptions were associated with subsequent PRTF admission?
(2) What types of child maltreatment findings are associated with subsequent PRTF admission?
(3) Are patterns of foster care placement, permanency, and return to care associated with subsequent PRTF admission?
(4) Is a history of family or child-only participation in TANF associated with subsequent PRTF admission?

We hypothesize that the hazard of PRTF admission is associated with greater behavioral and mental health needs, service utilization, and drug prescriptions. Further, we hypothesize that after adjusting for these factors, PRTF admission is associated with worse maltreatment outcomes; less stable foster care placement patterns, placements in treatment settings, and return to care after permanency; and participation in TANF.

## 2. Methods

### 2.1. Sample

The data used in this study come from administrative data files tracking reports of child maltreatment, foster care placements, family or child-only participation in TANF, and Medicaid claims from the state of North Carolina. Children in families first investigated for child maltreatment in North Carolina from 1 January 2007 to 31 July 2012, who had not been admitted to a PRTF prior to maltreatment, constituted the study population. From this population we sampled children between 5 and 17 years of age on 1 January 2007, and eligible for Medicaid during the study period. Preliminary descriptive analysis indicated that no children younger than age 5 entered a PRTF, suggesting that they were not part of the risk set. Choosing only youth eligible for Medicaid during the study period ensured that PRTF admission could be detected using Medicaid claims. The study period itself was determined by the availability of statewide Medicaid claims data. These constraints yielded a final analytic sample of 105,982 children.

### 2.2. Data Sources

A longitudinal data file was constructed that combined data from four sources: Medicaid claims, child abuse and neglect reporting, foster care, and income maintenance (TANF). The latter three sources were obtained from a database maintained by the School of Social Work at the University of North Carolina at Chapel Hill under a contract with the state Department of Health and Human Services. Linkages across systems were conducted using confidential identification numbers where possible. TANF and Medicaid data use the same identification number; and foster care and child maltreatment records use the same identification number. To connect these two identification numbers, we relied on a matching routine consisting of a series of rules connecting persons on names, social security numbers, and birthdates. Names are linked via a Soundex algorithm implemented using SAS version 9.3 (The SAS Institute, Cary, NC, USA); Social Security number and date of birth allow for one-digit errors and transposes of digits.

Dates and findings pertaining to child maltreatment reports from 1 January 2007 to 31 July 2012, as well as child date of birth, gender, and race/ethnicity, were obtained from statewide longitudinal child abuse and neglect system data. Foster care placement data from 1 January 2007 to 31 July 2012, including patterns of placement, types of placement during first spell, and exit from first spell, were obtained from a longitudinal file tracking all foster placements statewide. TANF participation data from 1 January 2007 to 31 July 2012 was obtained from a longitudinal file tracking all benefits paid to families and children across the state. Medicaid claims from 1 January 2007 to 31 July 2012 provided diagnosis, therapeutic services, residential care settings, psychotropic and antipsychotic drug prescription records, and PRTF admission data during this period. Two sources of claims data were used. For 85 counties, the state provided Medicaid claims records. For the remaining counties, a managed care organization (MCO) that operated under a waiver program provided the claims. Structural differences in the two sources was a limiting factor for the number and types of behavioral health variables that could be used. Data management and analysis were conducted in SAS 9.3. All study procedures, including data linkage and management, were approved by the University of North Carolina at Chapel Hill Institutional Review Board (13-3947; 2 November 2015).

### 2.3. Variables

#### 2.3.1. Demographic Covariates

Child age at first maltreatment report, gender (female), and race/ethnicity (Non-Hispanic Black, American Indian/Alaskan Native, Asian, Native Hawaiian/Pacific Islander, and White; and Hispanic ethnicity), were included as demographic covariates. A set of indicator variables for year of investigation were included to capture the changing risk of PRTF admission as youth enter the sample (due to first reported maltreatment investigation) closer to the study end date of 31 July 2012.

#### 2.3.2. Independent Variables

Each of the four sources of data provided potentially important predictors of PRTF admission that were central to answering the exploratory research questions. Each of the predictors was coded as a binary variable. Youth experiencing the characteristic on or after 1 January 2007 and prior to PRTF admission or the end of the study (for those youth not admitted by 31 July 2012) were coded a “1” and those youth not experiencing the characteristic prior to admission or end of study were coded a “0”. From Medicaid claims data, we used International Classification of Diseases, Ninth Revision (ICD-9) codes to identify youth with a trauma-associated behavioral health diagnosis including primarily mental disorders ([16]; the full list of ICD-9 codes and descriptions is available in Supplemental Table S1); billing provider type code to identify behavioral health services utilization (family, group, and individual therapy; care in less restrictive residential service settings; psychiatric services; and drug/alcohol services); and drug class numbers to identify psychotropic and antipsychotic drug prescriptions. From child abuse and neglect reporting data we obtained maltreatment substantiation (abuse, neglect, abuse and neglect, dependency, and services needed). From foster care placement records we obtained first placement date; placement in at least one group home, therapeutic home, treatment center, or kinship care during the first foster care spell; exit from first spell to permanency; and return to care after permanency placement. From income maintenance data, we identified youth from families receiving TANF benefits and youth receiving child-only benefits.

#### 2.3.3. Dependent Variable: Time to PRTF Admission

We used the Medicaid claims data to create a dummy variable identifying youth admitted to a PRTF (1 = admitted, 0 = not admitted or censored). PRTF records were identified by the billing provider type code. Less than 2% of youth in the sample were admitted to a PRTF during the study period (*n* = 1646). We also counted the number of days between the first maltreatment investigation to PRTF admission or censoring due to reaching the end of study or aging out at 18. It is possible that some of these youth could have been admitted to a PRTF prior to their first maltreatment. Although this is highly dependent on the age at first maltreatment given how few children under 5 were admitted, we cannot know the full extent to which this occurs or the effect on hazard ratios.

### 2.4. Analysis

Table 1 reports univariate statistics as well as a bivariate comparison of children admitted to PRTFs during the study period with those who were not (chi-square tests for binary variables and an independent sample *t*-test for age; Table 1).

#### 2.4.1. Event history Analysis

Cox proportional hazards (CPH) modeling was used to regress the time to PRTF admission on independent variables and covariates. In the CPH model, the baseline hazard of PRTF admission—which cannot be known—can be ignored because it cancels out of the function prior to estimation and thus no underlying distributional specification is needed [24]. Event history models (also known as survival and duration models) are finer-grained than logistic regressions of event occurrence, taking advantage of the longitudinal nature of the administrative data sources. In order to ensure the proper causal ordering—that youth characteristics putatively predicting admission do not occur after admission—we allow these characteristics to vary according to whether they occur before or after PRTF admission. For example if a youth is admitted to foster care after admission to a PRTF this youth would not be counted among those admitted to foster care. Similar to logistic regression, coefficient estimates are not typically directly interpreted, but are first transformed using exponentiation, in this case into hazard ratios. The hazard ratio describes the added hazard of PRTF admission for participants in the focal condition relative to the reference condition (or in the case of age, which is continuous, for a one-year difference in age). Time to event was measured as days from first maltreatment investigation to admission or censoring (for those not admitted before the end of study). SAS version 9.3 was used to estimate these models.

Like logistic regression, event history models do not have an error term and as a result unobserved heterogeneity may cause the hazard of PRTF admission to appear lower than it is. Hazard rates that appear to increase may be somewhat biased but are nevertheless increasing. Alternatively, decreasing or steady hazards may be, if sufficiently biased, increasing, constant, or decreasing. This can be detected using the conditional cumulative hazard curve for each model [25]. Ties in event times are not tractable within the partial likelihood function, but are handled by an approximation procedure [25]. We used the exact procedure, which is the most computationally intensive but best approach.

Censoring and truncation are the major issues that must be addressed when using event history models. Right censoring occurs when youth who do not experience PRTF admission prior to 31 July 2012 remain at risk of experiencing it after this date, even though we cannot detect it. In addition, youth who leave the state or leave Medicaid before the end of study are right censored as well. Only youth who remain in the state, remain on Medicaid through age 18, and reach adulthood without being admitted can be said to have definitely not experienced PRTF admission. Right censoring is only a limitation if it is associated with admission to a PRTF (i.e., informative) [25]. We have attempted to address left censoring and truncation by narrowly defining the population to those youth 5–17 years old on 1 January 2007 who are first maltreated within the study period who have not previously entered a PRTF. This is intended to address left censoring by ruling out youth who previously entered a PRTF, and to address left truncation by ensuring that youth only enter the risk set after the first observed maltreatment report. However, it is possible that some youth (more likely older ones) may have been admitted to a PRTF prior to 1 January 2007 and prior to their first maltreatment report. We would not be able to detect this prior event and would have included these youth in the sample.

#### 2.4.2. Model Fitting

We estimated a number of different CPH models, using an ordered process to help discern sources of shared variance that the independent variables may have had with each other as well as with the covariates. First, we used bivariate models of each independent variable to estimate unconditional hazard rates. Second, we estimated a model with all independent variables together. Finally, we estimated a model combining the independent variables and covariates. The Akaike Information Criterion (AIC) was used as an indicator of relative fit. Without predictors, the AIC was 24,529.541; better fit is indicated by lower values.

#### 2.4.3. Missing Data

The sample was obtained from a data file consisting of maltreatment investigations. Although most of these youth (96%) had a Medicaid claim during the study period, only 3.5% were present in the foster care data and only 16% were in the TANF data file. In these situations, we assumed the youth did not have the characteristics associated with those data sources. For example, if the youth did not have a foster care record, then we assumed that youth had no foster care placement. We therefore imputed zeros wherever there was a missing value. Some of these children may have been in the foster care or income maintenance systems of other states at some point during the study period, and in either case, the likelihood of having received Medicaid in that state was high.

## 3. Results

### 3.1. Sample Description and Bivariate Tests

The sample was 51.1% female; 36.2% Black; 46.8% White; 1.5% American Indian/Alaskan Native; 0.6% Asian; 0.2% Native Hawaiian/Pacific Islander; 4.1% other non-Hispanic; and 10.7% of Hispanic ethnicity (Table 1). The average age at first maltreatment report was just over 11.5 years; the average age at the start of the study was just over 9 years. 

The sample contained only a small proportion of youth (*n =* 1646; 1.6%) admitted to a PRTF during the study period. The median length of stay was just over 6 months. Prior to admission or censor, 41.5% were given a diagnosis that was associated with some type of trauma. Behavioral health service utilization prior to admission or censor ranged from 0.2% for psychiatric services to 23.7% for individual therapy. Regarding prescriptions, 30.1% of youth were prescribed at least one psychotropic drug; 18.8% prescribed at least 2; and 11.8% at least three. Antipsychotic drugs were prescribed to 10% of the sample. Prior to admission or censor 1.5% of youth experienced abuse, 7.9% neglect, 1.4% abuse and neglect, and 0.5% dependency. Prior to admission or censor, 3.5% had their first foster care placement and 2.4% returned to permanency. By type of placement in first spell, 1.2% were placed in group homes; 1.4% in kinship care; 0.7% in therapeutic care; and 0.4% in a treatment center. TANF histories before admission or censor included 6.9% of youth with child only and 12.8% with family benefits.

The bivariate results show that youth admitted to PRTFs were significantly more likely, prior to admission, to have a trauma-associated behavioral health diagnosis; significantly more likely to have some behavioral health service utilization; significantly more likely to have been prescribed one, two, or three psychotropic drugs or an antipsychotic drug; significantly more likely to have experienced neglect, abuse and neglect, and dependency (but not abuse alone); significantly more likely to have been in their first foster care spell, returned to foster care after permanency, and placed in group, kinship, therapeutic, and treatment center settings at least once during their first foster care spell (but not to have exited foster care); were significantly less likely to have been in a family receiving TANF benefits (but not child-only benefits); were significantly less likely to be female, Black, American Indian/Alaskan Native, Asian, and Hispanic; were significantly more likely to be White; and were older both at first maltreatment and at the start of the study period.

### 3.2. Cox Regression Results

Models 1.1–1.20 consisted of 20 separate models for each of the independent variables (Table 2). Due to some youth having negative values for days to PRTF admission, 530 records were discarded and only 105,452 were used in the regression models. The cumulative hazard functions for these models were decreasing over time. The AIC ranged from 21,563.697 (for prescribed one or more types of anti-psychotic) to 24,530.745 (for exited from foster care). Consistent with the patterns shown in the bivariate comparisons, nearly all of the independent variables were significantly associated with increased hazard of admission and many had very large effect sizes (high hazard ratios). The exceptions were exit from foster, which was not significant; and being in a family or child-only case that received TANF assistance, for which youth had reduced hazard of PRTF admission.

Model 2 combined all of the predictors from Models 1.1–1.20. The cumulative hazard functions for this model were decreasing over time; the AIC was 20,540.956, indicating improvement over all of the bivariate models. The findings of this model suggest that many of the effects of the behavioral health, maltreatment, foster care, and TANF characteristics overlapped with each other, sharing variance, and potentially suppressing the effects of other predictors. Exit from foster care, which had previously not been significant in the unconditional model, was associated with reduced hazard of PRTF admission (HR = 0.1, *p* < 0.001), suggesting a suppression effect from some combination of the other predictors. In contrast to the unconditional model, placement in a therapeutic home during the first spell was associated with a lower hazard of admission (HR = 0.6), suggesting a suppression effect —which was strong enough to reverse the direction of association—was present for this characteristic as well.

In model 3 we added the covariates. The cumulative hazard was constant over time. The AIC was 20,423.195, which indicates that this is the best fitting model. Consistent with model 2, youth had significantly (*p* < 0.001 except as noted) higher hazards of PRTF admission on several characteristics, and these hazard ratios did not vary substantively with the addition of demographic covariates. Youth had higher hazard of admission if prior to admission they had a trauma-associated behavioral health diagnosis (HR = 3.9); spent time in a residential care setting (therapeutic family and group foster placement, HR = 1.5; residential treatment, HR = 4.2; secure residential treatment, HR = 1.7); received one or more types of antipsychotic drugs (HR = 8.7); were placed in foster care for the first time during the study period (HR = 1.9, *p* < 0.01) or returned to care during the study period (HR = 4.1); or were placed in a treatment center setting during the first spell in foster care (HR = 3.1). Exit from foster care (HR = 0.1, *p* < 0.001), being in a family or child-only case receiving TANF assistance (HR = 0.6, *p* < 0.001), and placement in a therapeutic home during the first foster care spell (HR = 0.6, *p* < 0.01) were associated with lower hazards of admission that did not vary substantially from what they were in model 2 prior to the addition of covariates.

## 4. Discussion

Although often appropriate for severe concerns and short-term stabilization, PRTFs are restrictive and costly settings for children to receive behavioral health services. For children in families investigated for maltreatment and who enter foster care, PRTFs can also be a setting for out-of-home care. Determining whether PRTF entry is associated with clinical need, or other factors, is important to understanding this component of the continuum of care. In this study, we build on previous work to expand on our knowledge of the predictors of PRTF admission among youth whose families have been investigated for child maltreatment [23]. The analysis used longitudinal data—limiting predictors to those that occurred prior to admission for those admitted—and a set of bivariate and multivariate event history regression models to answer four important questions towards understanding the pathways of PRTF admission among this vulnerable group.

To briefly summarize the key findings, first, we found that the following indicators of need were associated with greater risk for subsequent PRTF entry: a trauma-associated behavioral health diagnosis; receipt of care in a less restrictive service setting; and one or more antipsychotic drug prescriptions. Second, conditional upon a set of demographic covariates, and behavioral health, foster care, and TANF characteristics, a history of substantiated maltreatment was not associated with admission to a PRTF. A third set of findings was related to foster care placement, stability, and exit to permanency and PRTF entry, and some of these were large as well. Finally, we found that TANF participation as part of a family or as a child-only case was associated with lower hazard of PRTF admission, conditional on all other variables. Taken together, these findings suggest that among the variables examined in this study for this sample, the main driver of PRTF entry was mental health need, but that other social service factors and particularly CPS-related factors, are independently associated with admission after accounting for this need. Further, there were important suppression effects operating on several predictors including exit from foster care and placement in a therapeutic home during the first foster care spell.

### 4.1. Limitations

There are several limitations to the findings of the present study. First, as noted, censoring is an issue with event history models—the youth not entering a PRTF by 31 July 2012 could have entered one later; further, despite the care taken to narrowly define the population with respect to the study period, some youth may have had spells in a PRTF prior to 1 January 2007, then subsequently been maltreated for the first time, which disqualifies them from the population. There is no way for us to know to whom this may have happened so they may be included in the sample anyway. Further, some of the youth may have experienced the event or the predictor characteristics in a different state, or while receiving another form of health insurance and not Medicaid. If informative, this could bias the results [25,26].

Second, we cannot make causal inferences from these findings. We used the longitudinal data to ensure that admission did not precede the predictor, a necessary temporal ordering for causal inference. Nevertheless, each of these characteristics may have been a common correlate of other unmeasured contemporaneous variables, which means the design is insufficient for making causal inferences. Third, administrative data, despite their richness, are not constructed for the purpose of research and have reliability and validity concerns: only youth who were eligible for Medicaid had valid behavioral and mental health measures; the maltreatment, foster care, and TANF history measures may undercount youth who have histories outside of the state in question; and overall the accuracy of the data depends on how accurately the data were entered at the point of contact with the client and how consistently they were entered across each of the counties in the state and over the duration of the study period.

### 4.2. Implications

These findings have important policy and practice implications. First, our findings strongly suggest that clinical need plays a crucial role in explaining PRTF admission. This suggests that primarily youth with behavioral health needs, specifically needs related to trauma, are admitted to PRTFs. This is reflected in the hazards of admission associated with several behavioral and mental health measures (and the lack of association between most of the non-clinical independent variables.) These hazard ratios, some of which are very large (e.g., above 3.0), together indicate that children with these indicators of higher need are more likely to enter a PRTF later, compared to their peers without these indicators. For policymakers seeking to reduce PRTF entry, improving the availability of trauma-informed services in less restrictive settings may be an important component of the service array.

Importantly, policies governing the child welfare, behavioral health, and educational sectors of the system of care clearly state that children should only be placed in a PRTF if all other available less restrictive and home- and community-based options have been exhausted. Therefore, there is a great interest from an advocacy and policy standpoint to prevent unnecessary PRTF entry. Understanding the factors that predict admission and those that can act as leverage points to prevent admission is critical to this objective. Towards this end, we expanded the scope of our inquiry beyond direct measures of clinical need to incorporate child maltreatment, foster care, and income maintenance measures. Arguably these non-clinical factors may be potential leverage points or they may actually be indirect measures of clinical need, and further research is needed to flesh this out.

#### 4.2.1. Need, Predisposing Factors, and Enabling Factors

Importantly, maltreatment substantiation alone (e.g., independent of need) is not associated with the risk of PRTF entry after accounting for clinical need. This would be a reassuring finding for policymakers concerned that substantiated maltreatment alone is sufficient to increase risk for PRTF entry.

However, findings from this study suggest that among children who enter foster care, placement in a therapeutic home may reduce the risk for PRTF entry after accounting for clinical need. This may indicate that providing appropriate foster care services may be important to reducing the need for higher levels of care. Other types and levels of mental health services were not protective against PRTF entry. In fact, out-of-home care in residential treatment was strongly predictive of PRTF entry. These placements are likely proxy indicators of unmeasured need for services. Other indicators of need—a trauma-related diagnosis and record of antipsychotic prescription drug—were also strong predictors of PRTF entry. Placements characterized by a lower need for behavioral and mental health services, such as kinship care (e.g., [21]), were not predictive of admission to a PRTF when other characteristics were taken into account.

Findings indicate that children who do not exit foster care through permanency, and then experience subsequent placement instability, are more likely to enter a PRTF. Placement instability may be related to behavioral needs beyond what we have measured, therefore increasing the need for PRTF services. However, it is also possible that PRTF entry for children in foster care could be related to availability of providers equipped to support children with complex behavioral needs. Future research is needed to understand how the service environment interacts with child need in predicting the level of care a child receives.

The association between TANF receipt and admission requires further research. However, there are two possibilities to explore. First, if TANF receipt is considered a proxy indicator for low-income status, it is possible that this finding suggests service setting is associated with child socio-economic status. We found that African-American and Native-American children had lower risk for admission. Therefore, it is possible that children from underserved groups (i.e., low-income, race/ethnic minority) are more likely to receive behavioral health services in other settings (i.e., juvenile justice), or not receive services at all. Further exploration is needed to examine the hypotheses generated from this finding.

#### 4.2.2. Unmeasured Factors

It should be mentioned that the aforementioned associations may be wholly or partly related to unmeasured predictors of PRTF admission. As noted, there were differences between bivariate and multivariate models, thus suggesting relationships among the independent variables. One implication for future research is to better understand the confounding, mediating, and suppressing nature of the various characteristics studied as predictors of PRTF admission; and to identify and measure additional predictors of admission. The low and non-significant hazard associated with less restrictive care settings, for example, is an average for all youth in the sample served in these settings, some of whom may have benefited but whose need was potentially much greater than those who did benefit. An important implication of these findings is to obtain a more refined and robust measure of need.

#### 4.2.3. Low Preadmission Service Utilization

Although our main finding provides some indication that need is the main driver of placement in restrictive care, there are several findings that give some cause for concern and present opportunities for future research. First, the level of service use prior to PRTF entry was higher for children who subsequently entered a PRTF. However, only half of these children received group therapy and about two-thirds received individual therapy prior to PRTF entry. A higher proportion of these children received pharmaceutical intervention (psychotropic and antipsychotic drugs). Although the use of therapeutic and pharmaceutical interventions was much higher for children who entered a PRTF compared to those who did not (at least as indicated in Medicaid claims records), one would possibly expect that all of the children who entered a PRTF would have received other services prior to PRTF entry.

A study of interviews with 88 private and public child welfare agency workers identified a number of concerns regarding the prevalence of pharmacology, lack of continuity in psychiatric treatment, lack of involved parents or guardians for youth serviced by the child welfare system, and a skepticism in the community of child welfare professionals towards the psychiatry profession [27]. These results suggest that in fact even among youth who are most likely to need the types of behavioral and mental health services that PRTFs provide, few of them are actually receiving the services prior to PRTF admission that could potentially prevent a costly and restrictive placement in such a setting. As a placement of last resort, more research is needed to determine why some children who enter a PRTF do not have access to such services prior to entry.

#### 4.2.4. Racial and Ethnic Disparities

An additional finding from this study that provides a direction for future research is the disproportionality in PRTF entry by race/ethnicity. Specifically, white children appear to be over-represented in the PRTF group (59%) compared to their proportion in the sample of children investigated for maltreatment (47%). Further, in the final multivariate model controlling for a host of covariates, African-American and American-Indian/Alaska Native children were significantly less likely to enter a PRTF compared to white children. This finding is consistent with other research identifying disparities in access to quality mental health services among racial/ethnic minority children [28,29]. Other studies suggest that among children with similar levels of mental health need and behaviors, racial/ethnic minority children are more likely to be referred to juvenile justice system settings compared to white counterparts [6,30]. Other studies suggest that differences in the recognition of mental health problems and subsequent help-seeking behaviors may different among caregivers of minority children [31,32].

#### 4.2.5. Big Data

Finally, a key implication of this study is the value of linked administrative data sources—a type of “big data” for social services. Youth served by public systems of care, particularly CPS, have higher mental health service utilization [33]. Surveys (e.g., NSCAW) have provided invaluable information and have made use of interview data and psychometrically validated tools to measure behavioral problems. However, the stylized nature of samples drawn for these types of studies (whether randomized or more convenient), and problems with participants’ recall of past events make it difficult to estimate the population trends in need and service utilization and estimate unbiased coefficients. Administrative data are already collected and can be leveraged, for a fraction of the cost of a representative sample study, to provide insight into the relationships between the typical markers of well-being across a variety of public support domains and admission to a PRTF.

## 5. Conclusions

Psychiatric residential treatment facilities (PRTFs) are highly restrictive settings for treating children with behavioral health needs, a setting that comes with substantial cost relative to community settings. Preventing unnecessary stays in PRTFs is therefore a worthwhile policy goal. These results show that there is an association between behavioral health needs and PRTF admission, and that certain types of foster care placements such as therapeutic home placement reduce children’s risk for PRTF admission, which is consistent with this goal. However, other factors that are not directly related to need, such as foster care placement stability and permanency, are also associated with PRTF admission. These likely constitute some combination of unmet needs, unmeasured correlates of PRTF admission, and the behavioral health risks associated with placement instability. Less clear is the role of family income status, race, and other socioeconomic factors. In both cases, more research is needed, and this research will benefit from linked administrative data.

## Figures and Tables

**Table 1:** Description of the study sample with bivariate analyses comparing children with and without later PRTF admission.

Youth Characteristic	All		PRTF		No PRTF		Comparison and Test Statistic		*p*
	(*n =* 105,982)		(*n =* 1646)		(*n =* 104,336)				
	*n*	%	*n*	%	*n*	%	Unadjusted OR	Chi Square	
Trauma diagnosis	43,973	41.5	1460	88.7	42,513	40.7	11.41	1534.96	<0.0001
Family therapy	17,374	16.4	823	50.0	16,551	15.9	5.30	1377.75	<0.0001
Group therapy	1694	1.6	144	8.7	1550	1.5	6.36	543.46	<0.0001
Individual therapy	25,169	23.7	1130	68.7	24,039	23.0	7.31	1861.63	<0.0001
Therapeutic family and group foster placement	3639	3.4	561	34.1	3078	3.0	17.01	4736.80	<0.0001
Residential treatment	2832	2.7	697	42.3	2135	2.0	35.16	10,118.54	<0.0001
Secure residential treatment	282	0.3	95	5.8	187	0.2	34.11	1909.67	<0.0001
Drug/alcohol services	13,195	12.5	554	33.7	12,641	12.1	3.68	689.86	<0.0001
One or more types of antipsychotic	10,547	10.0	1232	74.8	9315	8.9	30.36	7857.73	<0.0001
One or more types of psychotropic	31,946	30.1	1385	84.1	30,561	29.3	12.81	2315.41	<0.0001
Three or more types of psychotropic	12,517	11.8	1094	66.5	11,423	10.9	16.12	4794.92	<0.0001
Two or more types of psychotropic	19,967	18.8	1303	79.2	18,664	17.9	17.44	3978.79	<0.0001
Substantiated abuse	1629	1.5	23	1.4	1606	1.5	0.91	0.22	0.642
Substantiated neglect	8396	7.9	153	9.3	8243	7.9	1.19	4.32	0.038
Substantiated abuse and neglect	1453	1.4	33	2.0	1420	1.4	1.48	4.97	0.026
Substantiated dependency	499	0.5	24	1.5	475	0.5	3.24	34.77	<0.0001
First placement in foster care	3677	3.5	159	9.7	3518	3.4	3.06	191.31	<0.0001
Exited from first foster care PA	2585	2.4	29	1.8	2556	2.4	0.71	3.22	0.073
Return to foster care after exit	152	0.1	10	0.6	142	0.1	4.49	25.15	<0.0001
First foster care spell includes group home	1302	1.2	131	8.0	1171	1.1	7.62	624.12	<0.0001
First foster care spell includes kinship care	1467	1.4	33	2.0	1434	1.4	1.47	4.72	0.030
First foster care spell includes therapeutic home	778	0.7	92	5.6	686	0.7	8.95	540.88	<0.0001
First foster care spell includes treatment center	396	0.4	109	6.6	287	0.3	25.71	1753.63	<0.0001
TANF child-only	7264	6.9	95	5.8	7169	6.9	0.83	3.07	0.080
TANF (Work First benefits)	13,546	12.8	119	7.2	13,427	12.9	0.53	46.23	<0.0001
Child is female	54,110	51.1	660	40.1	53,450	51.2	0.64	80.35	<0.0001
Race: Black	38,391	36.2	491	29.8	37,900	36.3	0.75	29.59	<0.0001
Race: American Indian/Alaskan Native	1464	1.4	7	0.4	1457	1.4	0.30	11.22	0.001
Race: Asian	687	0.6	3	0.2	684	0.7	0.28	5.64	0.018
Race: Native Hawaiian/Pacific Islander	182	0.2	5	0.3	177	0.2	1.79	1.70	0.192
Race: White	49,596	46.8	967	58.7	48,629	46.6	1.63	95.93	<0.0001
Ethnicity: Hispanic	11,308	10.7	73	4.4	11,235	10.8	0.38	68.19	<0.0001
**Age**	**Mean**	**SD**	**Mean**	**SD**	**Mean**	**SD**	**Difference (Admitted-Not)**	***t*** **Statistic**	***p***
Youth age at first maltreatment report	11.6	3.2	10.45	2.56	11.54	3.21	1.42	−17.88	<0.0001
Youth age on 1 January 2007	9.2	3.1	14.60	2.54	9.15	3.12	1.30	−16.89	<0.0001

PRTF: psychiatric residential treatment facility; OR: Odds Ratio.

**Table 2:** Bivariate and Multivariate Cox Regressions Predicting PRTF Admission (*n =* 105,452).

Youth Characteristic	Models 1.1–1.20: Bivariate				Model 2: Multivariate				Model 3: With Covariates			
	Est. (SE)	HR	95*%* CI	*p*	Est. (SE)	HR	95*%* CI	*p*	Est. (SE)	HR	95*%* CI	*p*
Trauma-associated diagnosis	3.0 (0.1)	19.6	(15.5, 24.8)	<0.001	1.4 (0.2)	3.9	(2.9, 5.3)	<0.001	1.4 (0.2)	3.9	(2.9, 5.3)	<0.001
Family therapy	1.8 (0.1)	5.8	(5.2, 6.5)	<0.001	0.0 (0.1)	1.0	(0.9, 1.1)	0.917	0.0 (0.1)	1.0	(0.8, 1.1)	0.502
Group therapy	1.8 (0.1)	5.9	(4.8, 7.2)	<0.001	0.0 (0.1)	1.0	(0.8, 1.2)	0.745	0.0 (0.1)	1.0	(0.8, 1.2)	0.825
Individual therapy	2.2 (0.1)	8.6	(7.6, 9.8)	<0.001	0.0 (0.1)	1.0	(0.9, 1.2)	0.667	0.0 (0.1)	1.0	(0.9, 1.2)	0.584
Therapeutic family and group foster placement	2.8 (0.1)	17.2	(15.2, 19.4)	<0.001	0.4 (0.1)	1.5	(1.3, 1.8)	<0.001	0.4 (0.1)	1.5	(1.3, 1.7)	<0.001
Residential treatment	3.4 (0.1)	29.9	(26.5, 33.6)	<0.001	1.3 (0.1)	3.8	(3.3, 4.4)	<0.001	1.4 (0.1)	4.2	(3.5, 4.9)	<0.001
Secure residential treatment	3.3 (0.1)	27.3	(21.3, 35)	<0.001	0.4 (0.1)	1.5	(1.2, 2)	0.001	0.6 (0.1)	1.7	(1.3, 2.3)	<0.001
Drug/alcohol services	1.5 (0.1)	4.5	(4, 5.1)	<0.001	−0.1 (0.1)	0.9	(0.8, 1.1)	0.293	0.0 (0.1)	1.0	(0.8, 1.1)	0.596
One or more types of antipsychotic	3.5 (0.1)	33.7	(29.3, 38.9)	<0.001	2.2 (0.1)	8.6	(7, 10.6)	<0.001	2.2 (0.1)	8.7	(7, 10.7)	<0.001
one or more types of psychotropic	2.8 (0.1)	17.3	(14.4, 20.7)	<0.001	0.3 (0.1)	1.3	(1, 1.7)	0.072	0.2 (0.1)	1.3	(0.9, 1.7)	0.116
Substantiated abuse/neglect/dependency	0.6 (0.1)	1.9	(1.6, 2.2)	<0.001	0.0 (0.1)	1.0	(0.9, 1.2)	0.856	0.0 (0.1)	1.0	(0.9, 1.2)	0.634
Services needed or recommended	0.1 (0.1)	1.1	(1, 1.3)	0.046	0.1 (0.1)	1.1	(1, 1.3)	0.132	0.1 (0.1)	1.1	(1, 1.3)	0.114
First placement in foster care	1.4 (0.1)	4.2	(3.6, 5)	<0.001	0.8 (0.2)	2.2	(1.3, 3.4)	0.001	0.7 (0.2)	1.9	(1.2, 3.1)	0.007
Exited from first foster care PA	−0.2 (0.2)	0.8	(0.6, 1.2)	0.386	−2.5 (0.3)	0.1	(0, 0.1)	<0.001	−2.4 (0.3)	0.1	(0.1, 0.2)	<0.001
Return to foster care after exit	1.7 (0.3)	5.5	(2.9, 10.2)	<0.001	1.4 (0.4)	4.1	(1.9, 9)	<0.001	1.4 (0.4)	4.1	(1.9, 9)	<0.001
First foster care spell includes group home	2.3 (0.1)	10.2	(8.5, 12.2)	<0.001	0.0 (0.3)	1.0	(0.6, 1.8)	0.938	0.1 (0.3)	1.2	(0.7, 2.1)	0.617
First foster care spell includes kinship care	0.7 (0.2)	1.9	(1.3, 2.7)	<0.001	−0.1 (0.2)	0.9	(0.6, 1.4)	0.798	−0.1 (0.2)	0.9	(0.6, 1.4)	0.704
First foster care spell includes therapeutic home	2.5 (0.1)	11.8	(9.5, 14.6)	<0.001	−0.5 (0.2)	0.6	(0.4, 0.9)	0.006	−0.5 (0.2)	0.6	(0.4, 0.9)	0.005
First foster care spell includes treatment center	3.4 (0.1)	29.4	(24.1, 35.8)	<0.001	1.2 (0.2)	3.2	(2, 5.2)	<0.001	1.1 (0.2)	3.1	(1.9, 5.1)	<0.001
TANF	−0.5 (0.1)	0.6	(0.5, 0.7)	<0.001	−0.5 (0.1)	0.6	(0.5, 0.7)	<0.001	−0.5 (0.1)	0.6	(0.5, 0.7)	<0.001
Child is female									0.0 (0.1)	1.0	(0.9, 1.2)	0.493
Age at first maltreatment report									0.0 (0.0)	1.0	(1, 1)	0.690
Race: Black									−0.2 (0.1)	0.8	(0.7, 0.9)	0.001
Race: American Indian/Alaskan Native									−1.5 (0.6)	0.2	(0.1, 0.7)	0.010
Race: Asian									0.1 (0.7)	1.2	(0.3, 4.6)	0.841
Race: Native Hawaiian/Pacific Islander									0.8 (0.4)	2.2	(0.9, 5.2)	0.086
Ethnicity: Hispanic									−0.2 (0.1)	0.8	(0.6, 1.1)	0.109
Year of first investigation 2008									0.3 (0.1)	1.3	(1.1, 1.6)	0.001
Year of first investigation 2009									0.6 (0.1)	1.8	(1.5, 2.2)	<0.001
Year of first investigation 2010									0.8 (0.1)	2.3	(1.9, 2.8)	<0.001
Year of first investigation 2011									1.1 (0.1)	3.1	(2.4, 4)	<0.001
Year of first investigation 2012									1.1 (0.3)	3.1	(1.8, 5.1)	<0.001
Model fit (AIC)	21,563.697–24,530.745				20,540.956				20,423.195			

Est: parameter estimate; SE: standard error; TANF: Temporary Assistance to Needy Families; PA: placement authority; AIC: Akaike Information Criterion.

## Supplementary Materials

The following are available online at www.mdpi.com/1660-4601/14/10/1141/s1, Table S1: ICD-9 Behavioral Health Diagnoses Associated with Trauma.
