# Supplementary material for: A Longitudinal Study of Child Maltreatment and Mental Health Predictors of Admission to Psychiatric Residential Treatment Facilities
Source: Int J Environ Res Public Health. 2017 Sep 28;14(10):1141. doi: 10.3390/ijerph14101141 (PMC5664642; doi:10.3390/ijerph14101141)
Supplement: Supplementary file 1 [file ijerph-14-01141-s001.pdf]

Table S1. ICD-9 Behavioral Health Diagnoses Associated with Trauma

ICD9	Description
V61.12	Physical Abuse of Adult (if by partner)
V61.12	Sexual Abuse of Adult (if by partner)
V61.20	Parent-Child Relational Problem
V61.21	Neglect of Child
V61.21	Physical Abuse of Child
V61.21	Sexual Abuse of Child
V62.3	Academic Problem
V62.83	Physical Abuse of Adult (if by person other than partner)
V62.83	Sexual Abuse of Adult (if by person other than partner)
293.83	Mood Disorder Due to...[Indicate the General Medical Condition]
293.84	Anxiety Disorder Due to...[Indicate the General Medical Condition]
296.2	Major Depressive Disorder, Single Episode, Unspecified
296.21	Major Depressive Disorder, Single Episode, Mild
296.22	Major Depressive Disorder, Single Episode, Moderate
296.23	Major Depressive Disorder, Single Episode, Severe without Psychotic Features
296.24	Major Depressive Disorder, Single Episode, Severe with Psychotic Features
296.25	Major Depressive Disorder, Single Episode, In Partial Remission
296.26	Major Depressive Disorder, Single Episode, In Full Remission
296.3	Major Depressive Disorder, Recurrent, Unspecified
296.31	Major Depressive Disorder, Recurrent, Mild
296.32	Major Depressive Disorder, Recurrent, Moderate
296.33	Major Depressive Disorder, Recurrent, Severe without Psychotic Features
296.34	Major Depressive Disorder, Recurrent, Severe with Psychotic Features
296.35	Major Depressive Disorder, Recurrent, In Partial Remission
296.36	Major Depressive Disorder, Recurrent, In Full Remission
296.9	Mood Disorder NOS
300	Anxiety Disorder NOS
300.02	Generalized Anxiety Disorder
300.3	Obsessive-Compulsive Disorder
300.4	Dysthymic Disorder
308.3	Acute Stress Disorder
309	Adjustment Disorder with Depressed Mood
309.21	Separation Anxiety Disorder
309.24	Adjustment Disorder with Anxiety
309.28	Adjustment Disorder with Mixed Anxiety and Depressed Mood
309.3	Adjustment Disorder with Disturbance of Conduct
309.4	Adjustment Disorder with Mixed Disturbance of Emotions and Conduct
309.81	Posttraumatic Stress Disorder

309.9	Adjustment Disorder Unspecified
311	Depressive Disorder NOS
312.81	Conduct Disorder, Childhood Onset Type
312.82	Conduct Disorder, Adolescent Onset Type
312.89	Other Conduct Disorder
313.89	Reactive Attachment Disorder of Infancy or Early Childhood
313.9	Disorder of Infancy, Childhood, or Adolescence NOS
314	Attention-Deficit/Hyperactivity Disorder, Predominantly Inattentive Type
314.01	Attention-Deficit/Hyperactivity Disorder, Combined Type
314.01	Attention-Deficit/Hyperactivity Disorder, Predominantly Hyperactive-Impulsive Type
314.9	Attention-Deficit/Hyperactivity Disorder NOS
315	Reading Disorder
315.32	Mixed Receptive-Expressive Language Disorder
317	Mild Mental Retardation
318	Moderate Mental Retardation
318.1	Severe Mental Retardation
318.2	Profound Mental Retardation
319	Mental Retardation, Severity Unspecified
995.52	Neglect of Child (if focus of attention is on victim)
995.53	Sexual Abuse of Child (if focus of attention is on victim)
995.54	Physical Abuse of Child (if focus of attention is on victim)
995.81	Physical Abuse of Adult (if focus of attention is on victim)
995.83	Sexual Abuse of Adult (if focus of attention is on victim)
